# Single‐Cell Encapsulation via Click‐Chemistry Alters Production of Paracrine Factors from Neural Progenitor Cells

**DOI:** 10.1002/advs.201902573

**Published:** 2020-03-05

**Authors:** Byeongtaek Oh, Vishal Swaminathan, Andrey Malkovskiy, Sruthi Santhanam, Kelly McConnell, Paul M. George

**Affiliations:** ^1^ Department of Neurology and Neurological Sciences School of Medicine Stanford University Stanford CA 94305 USA; ^2^ Biomaterials and Advanced Drug Delivery Laboratory School of Medicine Stanford University Stanford CA 94305 USA

**Keywords:** ADCY8‐cAMP, extracellular matrix, glycoengineering, mechanotransduction, nonviral cell modulation, single‐cell encapsulation, stem cell therapies

## Abstract

Extracellular matrix (ECM) properties affect multiple cellular processes such as cell survival, proliferation, and protein synthesis. Thus, a polymeric‐cell delivery system with the ability to manipulate the extracellular environment can act as a fundamental regulator of cell function. Given the promise of stem cell therapeutics, a method to uniformly enhance stem cell function, in particular trophic factor release, can prove transformative in improving efficacy and increasing feasibility by reducing the total number of cells required. Herein, a click‐chemistry powered 3D, single‐cell encapsulation method aimed at synthesizing a polymeric coating with the optimal thickness around neural progenitor cells is introduced. Polymer encapsulation of neural stem cells significantly increases the release of neurotrophic factors such as VEGF and CNTF. Cell encapsulation with a soft extracellular polymer upregulates the ADCY8‐cAMP pathway, suggesting a mechanism for the increase in paracrine factors. Hence, the described single‐cell encapsulation technique can emerge as a translatable, nonviral cell modulation method and has the potential to improve stem cells' therapeutic effect.

## Graphical abstract

Through the use of cell glycoengineering techniques and click chemistry, single‐cell polymer encapsulation is possible. This polymeric extracellular matrix can modify the trophic factor production of neural progenitor cells. The cyclic adenosine monophosphate pathway and actin interactions are important in trophic factor regulation, specifically for VEGFB. The ability to encapsulate cells has implications for optimizing stem cell therapeutics.

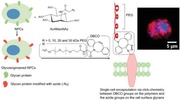

## 1 Introduction

Stem cell therapy is a promising treatment option for patients with cardiac, orthopedic, and neurological diseases.^[^ ^1^, ^2^, ^3^, ^4^ ^]^ Several clinical trials have demonstrated the successful and safe delivery of stem cells in the target region and their ability to improve functional outcomes.^[^ ^5^, ^6^, ^7^ ^]^ However, there is still a need to improve the therapeutic efficacy, especially for neural deficits, and to prolong cell survival in the often harsh target environment. Moreover, often hundreds of millions of stem cells are transplanted to obtain a beneficial therapeutic effect, thereby increasing the time, cost, and risk associated with these procedures.^[^ ^8^, ^9^ ^]^ To overcome the limitations, polymer‐mediated cell encapsulation methods have been applied to therapies aimed at restoring injured tissue, particularly in central nervous system disorders.^[^ ^10^, ^11^ ^]^

Biomaterials have been extensively explored to deliver neural stem cells and to promote robust cell growth, survival, and trophic factor release.^[^ ^12^, ^13^ ^]^ This has led to a myriad of developments, such as programmable and injectable hydrogels serving as cell scaffolds.^[^ ^14^, ^15^, ^16^ ^]^ In order to synthesize these commonly employed vehicles, building blocks such as hyaluronic acid or chitosan are chemically or physically cross‐linked often with azide‐alkyne bonds or ionic interactions.^[^ ^17^, ^18^ ^]^ These biomaterials and methods for encapsulation have yielded results of varying degrees of success, ranging from preclinical development to advanced clinical trials.^[^ ^19^, ^20^ ^]^ However, a limitation in current methods of cell encapsulation lies in the fact that there is often a plateau in regards to the maximal therapeutic effect derived from a single transplant. A uniform method of cellular encapsulation could produce more reliable and effective results. Glycoengineering, a technique that allows manipulation of cellular membrane glycans to modify cells, provides an intriguing method to homogenously regulate paracrine properties at a cellular level.^[^ ^21^, ^22^, ^23^, ^24^ ^]^

In this work, we developed a single‐cell encapsulation method via click‐chemistry and glycoengineering. This technique creates an efficient way to coat a layer of polymer around each neural progenitor cell (NPC). By varying the stiffness of the polymer coating, we were able to modulate the proteins released by the cells. The optimized tactile interactions with the polymeric coating surrounding the cells enhance trophic factor release, such as vascular endothelial growth factor (VEGF). By augmenting the therapeutic benefit of each NPC, the number of cells needed to cause a therapeutic effect in a biological system can be reduced.

## 2 Results and Discussion

### 2.1 Construction and Optimization of Click‐Chemistry Powered Glycoengineering

The stiffness of the extracellular matrix (ECM) can vary greatly with the extremes seen in pathologic conditions such as cancer and glioma.^[^ ^25^, ^26^ ^]^ Based on previously established studies, the stiffness of the surrounding cell environment, as defined by this relationship between stress and strain using Young's Modulus, plays a critical role in dictating cellular function, proliferation, and survival.^[^ ^27^, ^28^, ^29^ ^]^

**Figure 1:**
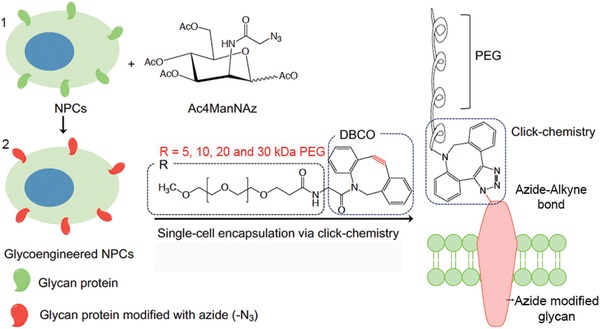
Schematic illustration of the single‐cell encapsulation of NPCs via click‐chemistry. 1) NPCs are treated with Ac4ManNAz to produce azide modified cells. 2) NPCs expressing glycan protein modified with azide (—NH_3_) are encapsulated with varying molecular weights of PEG (R) via a click crosslinking reaction between the azide group and DBCO modified PEGs with the alkyne group.

**Figure 2:**
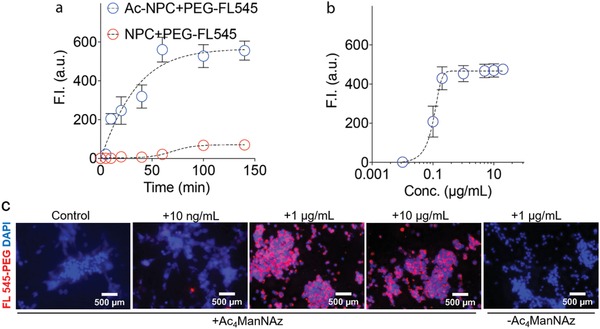
Optimization of cell encapsulation parameters. a,b) Optimization of the concentration and time parameters for effective homogenous and individual coating of cells with the PEG polymer. Maximization of the fluorescent intensity (F.I.) seen due to the FL545 moiety of the coated PEG polymer corroborates this optimization. c) IFC images illustrate the relationship between the various concentrations of the DBCO‐PEG‐FL545 and the resulting illumination. Control is the cells without encapsulation (+Ac_4_ManNAz, 0 ng mL^−1^ polymer).

**Figure 3:**
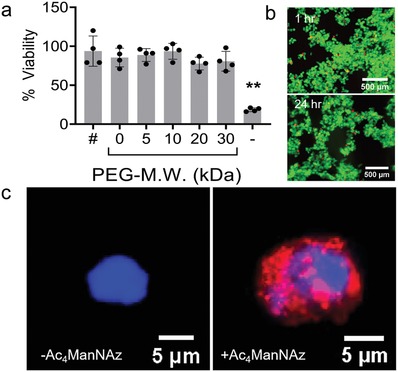
Single‐cell encapsulation. a) Viability of PEG coated NPCs. The percentage viability is normalized to the positive control (cells alone without Ac_4_ManNAz or polymer encapsulation). # is the cell treated with 30 kDa PEG without prior treatment with Ac_4_ManNAz and – is the negative control, cells treated with cell lysis solution. All experimental groups, cell coated with varying M.W. of PEG, are viable without any significant difference compared to control. Analyzed using a one‐way ANOVA, followed by Tukey's HSD post hoc test with ** *p* < 0.01. b) Representative live/dead images demonstrate high viability of encapsulated cells with 30 kDa PEG. c) IFC image providing evidence of encapsulation of the cell with PEG‐FL545 coating (right) compared to the control (left). The FL545 moieties contribute to the red fluorescence around the nucleus stained with DAPI (blue fluorescence).

To determine the optimal stiffness of the polymer coating for our single‐cell encapsulation technique, we evaluated dibenzocyclooctyne‐polyethyl glycol (DBCO‐PEG) chain coatings of various molecular weights (5, 10, 20, and 30 kDa) attached via click‐chemistry (**Figure** **1**). By varying the molecular weight of the polymer attached to the NPCs, the stiffness of the immediate environment surrounding the cells could be altered, resulting in cellular control of differing capabilities and properties. First, we examined the required incubation time of cells incorporated with the Ac_4_ManNAz attachment moiety in media containing a FL545‐tagged DBCO‐PEG polymer. Subsequently, the ideal concentration of the DBCO‐PEG for maximal cellular encapsulation was evaluated. Based on the fluorescence intensity readings, we found that attachment of the polymer reached a maximal plateau beginning at ≈60 min of incubation (**Figure** **2**a) and 1 µg mL^−1^ of PEG in the media (Figure 2b). Thus, these parameters were utilized for the remainder of the experiments. Further, in the control groups, NPCs without the glycans modified with Ac_4_ManNAz (‐Ac_4_ManNAz) and cells with Ac_4_ManNAz but without the polymer (Control), a strong signal of FL 545‐PEG was not observed in the fluorescent microscopy images of cells compared to those coated with different concentrations of FL 545‐PEG (Figure 2c). This indicates the cells were only encapsulated by polymer if the Ac_4_ManNAz moiety was present. Interestingly, while growing in culture, the encapsulated cells appear to aggregate in a similar pattern as unencapsulated cells, in effect forming a multicellular nanoencapsulation.^[^ ^30^ ^]^ If cells are encapsulated individually through the click chemistry method even if some level of aggregation occurs at higher densities, single‐cell control would still be maintained by the immediate cellular environment provided by the single‐cell polymeric coatings. Next, the viability of cells encapsulated with varying molecular weights of PEG was determined. Encapsulation of the cells with the 5, 10, 20, or 30 kDa PEG experienced minimal cell death after incubation for 24 h (**Figure** **3**a,b), demonstrating the process is well tolerated by the NPCs.

To ascertain that individual cells were indeed being coated, the cells were visualized with fluorescent microscopy (BZ‐X710, Keyence, Itasca, IL) and transmission electron microscopy (TEM, JEM‐1400, JEOL solutions, Peabody, MA). High magnification images of fluorescently tagged PEG were obtained to verify a layer of polymer surrounding individual cells. The images reveal a layer of red fluorescence around the NPC with the Ac_4_ManNAz moiety, confirming a single‐cell nanoencapsulation with the FL 545‐PEG (Figure 3c). The TEM image of a 30 kDa PEG coated NPC sample shows a layer of different grayscale along the cell membrane (Figure S1, Supporting Information), which may suggest this layer of polymer coating around the cell.

### 2.2 Verification of NPC Modification by Encapsulation

**Figure 4:**
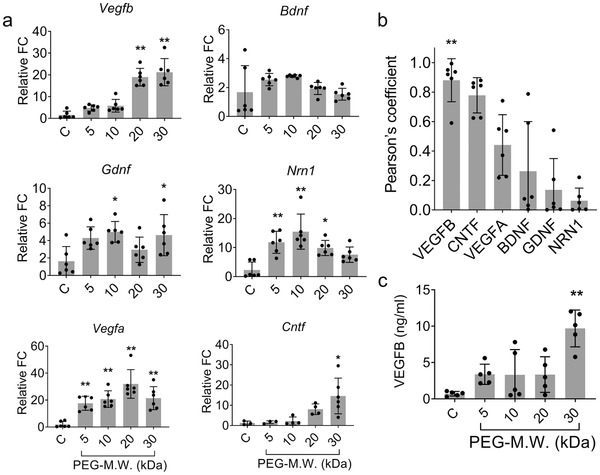
Different molecular weights of polymer encapsulation affect trophic factor release. a) Bar graphs portraying the trend of increasing transcription of neurotrophic factors (measured using qRT‐PCR) in relation to the various weights of the polymer chain used for encapsulation. The factor expression corresponding to each of the different polymer weights used is denoted in terms of fold change (F.C.) with respect to the control group (C), cells without encapsulation. b) Pearson's coefficient analysis is also provided to denote the correlation between proper encapsulation and increased factor release. c) ELISA results indicating increased VEGFB production with various molecular weights of DBCO‐PEG. a–c) Analyzed using a one‐way ANOVA, followed by Tukey's HSD post hoc test with ** *p* < 0.01 and * *p* < 0.05. In (b), VEGFB was statistically significant compared to other groups. In (c), 30 kDa polymer encapsulation was statistically different from other groups. Values represent the mean of independent experiments (*n* = 4); error bars, S.D.

Polymers have been shown to modulate the inherent mechano‐sensing properties of cells.^[^ ^31^ ^]^ To evaluate if the polymer modified the cellular properties of the NPCs, the transcription of trophic factors by the polymer‐encapsulated NPCs were evaluated using quantitative real‐time polymerase chain reaction (qRT‐PCR). It was observed that polymer encapsulation caused an increase in trophic factor transcription (**Figure** **4**a) compared to the control (C, cells without polymer encapsulation). The augmentation of factor release is clearly exemplified based on the fold increases in the release of various important neurotrophic molecules such as vascular endothelial growth factor A (VEGFA), vascular endothelial growth factor B (VEGFB), brain‐derived neurotrophic factor (BDNF), ciliary neurotrophic factor (CNTF), glial cell‐derived neurotrophic factor (GDNF), neuritin 1 (NRN1). For many factors, the coatings of higher molecular weight result in higher fold factor release. Both VEGFA and VEGFB showing the largest increases at almost 20‐fold change compared to the unencapsulated control group (Figure 4a). To confirm that varying the molecular weight of the polymer was indeed the variable accounting for the increased trophic factor release, Pearson's coefficients for different trophic factors were plotted based on the qRT‐PCR analyses. The Pearson's coefficients, a measure of linear correlation between trophic factor release and polymer encapsulation, confirm the effectiveness of our methods. All of the factors have a correlation greater than 0, with VEGFB having the strongest positive correlation with a coefficient of almost 0.88 ± 0.15 (Figure 4b). The factors with the highest Pearson's coefficients align with previous studies that show VEGF and CNTF respond to mechanical stretch.^[^ ^32^ ^]^ Notably, members of the neurotrophin family, BDNF and NRN1, had smaller Pearson's coefficients, indicating this family of proteins may be less responsive to mechanical stimuli.^[^ ^33^ ^]^

An enzyme‐linked immunosorbent assay (ELISA) study was conducted to measure the concentration of ELISA in the supernatant to determine if the gene modifications resulted in a change in protein concentration of VEGFB (the factor with the highest Pearson coefficient). The concentration of the factor released in the media isolated from the encapsulated cells is almost a factor of 10 greater in the 30 kDa group (9.7 ± 2.5 ng mL^−1^) compared to the control group (0.7 ± 0.3 ng mL^−1^) (Figure 4c, *p* < 0.01). Interestingly, VEGFB transcription was found to be significantly increased in both the 20 and 30 kDa groups, but the confirmatory protein testing showed a significant increase in only the 30 kDa group. This may be an indication that a higher threshold may exist for encapsulation to cause changes in production at the protein level. These studies further corroborate the fact that our technique effectively modulates factor release from NPCs.

### 2.3 Analysis of Changes in Tropic Factor Release due to Manipulation of Mechanical Cues

**Figure 5:**
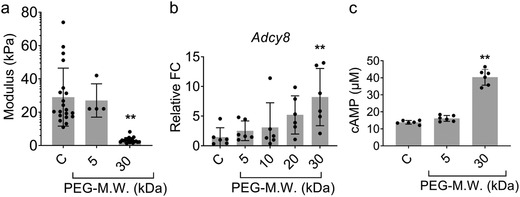
A softer layer of polymer encapsulation surrounding the cells augments ADCY8 expression and cAMP production. a) The AFM analysis illustrates that the encapsulation method modulates the Young's Modulus of the immediate environment surrounding the cells. Higher molecular weight PEG produces a softer polymeric ECM. b) qPCR‐RT results demonstrate the up‐regulation of ADCY8 with softer (higher molecular weight) PEG. c) Bioluminescent results show an increase in cAMP with softer PEG. a–c) C represents control group, which are cells without encapsulation. Analyzed using a one‐way ANOVA, followed by Tukey's HSD post hoc test with ** *p* < 0.01. 30 kDa group was statistically significant compared to other groups. Values represent the mean of independent experiments (*n* = 4); error bars, S.D.

To further investigate whether the increase in trophic factor production is associated with polymer mechanical characteristics, atomic force microscopy (AFM) was used to determine the stiffness of an individual NPC's surface modified with polymer. Based on the results from neurotrophic factor release, the experimental groups that were best representatives, including a control group without polymer, and NPCs modified with 5 kDa and 30 kDa PEG were chosen for this study. The Young's elastic modulus is a measure of a substance's ability to resist deformation. It is calculated by dividing the stress placed on the substance in question by the strain it experiences. The AFM technique was able to measure the Young's modulus of individual NPCs from the control, 5 kDa PEG, and 30 kDa PEG groups. Because the control group consists of cells alone, the Young's modulus is the measurement of stiffness from the cellular surface, which is primarily produced by the cytoskeleton, nucleus and other internal organelles.^[^ ^29^ ^]^ The neural progenitor cell stiffness was found to be about 20 kPa (**Figure** **5**a). Our measured stiffness falls within the wide range of stiffness observed in stem cells and neural cells from previous studies using AFM (range from 30 Pa to greater than 100 kPa).^[^ ^34^, ^35^ ^]^ Interestingly, the 5 kDa PEG‐coating had a similar stiffness to the cells alone (the control group). However, cell encapsulation with polymers of a larger molecular weight (30 kDa PEG) exhibit a smaller Young's modulus, indicating an environment that is softer than that of the 5 kDa PEG. Compared to the 5 kDa polymer groups, individually encapsulating NPCs with a polymer weighing 30 kDa resulted in an ECM with a stiffness lowered by almost a factor of 10 based on the Young's moduli measurements (Figure 5a). These results are consistent with previous findings that the stiffness of PEG decreases with higher molecular weights.^[^ ^36^ ^]^ Presumably, this is related to the fact that there is a change in crosslink density of PEG with varying molecular weight, thereby producing less stiff polymers with an increase in its molecular weight.^[^ ^37^ ^]^ In addition, The uniformity of each single cell that was probed indicates single‐cell nanoencapsulation results from this technique in these conditions. These results elucidate the ability of the cell encapsulation technique to modulate the NPCs' immediate surrounding environment.

In order to delineate which pathways may play a role in converting the mechanical signals into increased trophic factor release, we studied an important pathway in cell signaling, the cyclic adenosine monophosphate (cAMP) dependent pathway. Previous work demonstrated that cAMP signaling activated by mechanical stimuli is produced at the cell surface.^[^ ^38^ ^]^ cAMP is also well known to regulate cell paracrine factor expression.^[^ ^39^, ^40^, ^41^ ^]^ External mechanical cues can activate adenylyl cyclase, which catalyzes conversion of ATP to cAMP.^[^ ^42^ ^]^ Specifically, adenylate cyclase 8 (ADCY8) plays an important role in cAMP regulation.^[^ ^43^ ^]^ Thus, we analyzed ADCY8 and cAMP levels using qRT‐PCR and a luminometric assay (Figure 5b,c, respectively) to determine if they were altered by the properties of the PEG. Using the same representative groups as above, cells coated with softer PEG (30 kDa) upregulated ADCY8 as compared to the other groups. Additionally, cAMP conversion was very efficient in cells coated with softer PEG. These results suggest that cell encapsulation with the soft polymer alters mechanical stress‐induced cAMP signaling, resulting in the increased production of trophic factors. These findings support prior results demonstrating the upregulation of ADCY8 leads to higher production of cAMP.^[^ ^44^ ^]^

### 2.4 Inhibition Studies to Demonstrate Variation of ADCY8‐cAMP Mechanism during Mechanical Stimulation

**Figure 6:**
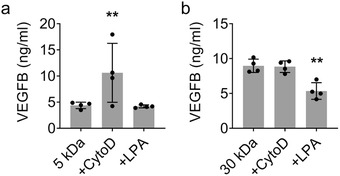
Inhibition and activation of actin polymerization alters trophic factor release. This trend is shown in cells encapsulated by both the 5 and 30 kDa polymers. a) Using cells encapsulated with 5 kDa PEG, VEGFB levels with CytoD treatment was significantly different from other groups with ELISA analysis. b) Using cells encapsulated with 30 kDa PEG, VEGFB levels with LPA treatment was significantly different from other groups with ELISA analysis. a,b) Analyzed using a one‐way ANOVA, followed by Tukey's HSD post hoc test with ** *p* < 0.01.Values represent the mean of independent experiments (*n* = 4); error bars, S.D.

Previous research shows that increasing the levels of cAMP in a cell lowers the levels of actin polymerization.^[^ ^45^ ^]^ One of the primary methods that a cell reacts to an increased stiffness of the ECM involves the actin cytoskeleton through actin dynamics.^[^ ^46^ ^]^ Because of actin's role in the cAMP pathway and mechanotransduction, we investigated the effect of inhibitors and activators of actin dynamics in response to the mechanical stimuli of PEG.^[^ ^47^ ^]^ Since VEGFB had been the trophic factor most largely effected by the mechanical properties of the coated polymer, we explored the role of actin and its effect on VEGFB release (**Figure** **6**).

Cytochalastin D (CytoD) inhibits actin polymerization.^[^ ^48^, ^49^ ^]^ Theoretically if the cAMP pathway was increased as seen in the soft PEG condition, CytoD would have less effect on trophic factor release (i.e., VEGFB) because the actin pathway would already be inhibited by cAMP upregulation. Indeed in our experiments comparing different polymer coatings, we found that CytoD increased VEGFB production in NPCs coated with 5 kDa PEG (Figure 6a). However, in NPCs coated with 30 kDa PEG, the VEGFB concentration was not significantly different, indicating that actin polymerization was already sufficiently inhibited by the softer polymer encapsulation.

**Figure 7:**
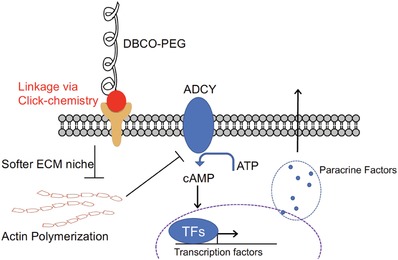
Graphical illustration of the proposed interplay between our novel click‐chemistry DBCO‐PEG single‐cell encapsulation system and actin polymerization, stressing how it affects the ACDY‐cAMP transduction pathway and factor release.

Given these results we hypothesized that an activator of actin would have the opposite effect. To further demonstrate this, lysophosphatidic acid (LPA, an activator of actin polymerization^[^ ^50^ ^]^) was applied to the encapsulated NPCs. Because the 30 kDa encapsulated NPCs inhibited actin polymerization, the reversal of actin inhibition through LPA results in decreased production of VEGFB (Figure 6b). Taken together, these results indicate that actin polymerization plays an important role in extracellular polymer regulated trophic factor release in the encapsulated cells (**Figure** **7**).

## 3 Conclusion

Polymeric cell encapsulation is an effective method to increase the survival and efficacy of cell transplantation.^[^ ^51^, ^52^, ^53^ ^]^ The development of a uniform nanoencapsulation technique described above allows for precise control of cellular trophic factor release by leveraging a cell's response to the extracellular polymer coating. While a polymeric cellular coating may differ from the ECM because of the complex interactions such as focal adhesions a cell has with the ECM, the intracellular pathways that react to cell membrane stiffness such as actin my cause similar changes in the cell. Further studies evaluating these differences are needed to elucidate further details of cellular mechanics. In addition to optimizing cellular function, the use of glycoengineering to form a consistent cellular encapsulation technique creates the opportunity to better understand the activity of stem cells at a cellular level. This understanding is essential to designing effective cellular modulation strategies and translational therapeutics.^[^ ^54^, ^55^ ^]^ Further modification of the polymer coating using this methodology could also be used to direct cellular attachments to other cells or surfaces, thereby paving a way for cellular/neural network reconstruction.

To conclude, by applying a new single‐cell encapsulation technique via click‐chemistry, we were able to investigate the effect of single‐cell encapsulation on trophic factor release. We discovered a feasible mechanism by which the molecular weight of the polymer controls cell surface stiffness and regulates cell signaling via modulation of the ADCY8‐cAMP pathway. Changes in ADCY8 and cAMP production due to mechanical properties of the polymers affect trophic factor release (specifically VEGFB) from cells, likely through the actin pathway. Further investigations are required to validate the extent of the mechanistic pathways suggested by our experiments. Our data demonstrates that through the use of the single‐cell encapsulation technique the properties of NPCs can be regulated and modulated by the polymer properties. Future studies examining this effect on biological systems will prove insightful into biomedical applications of this polymeric platform.

## 4 Experimental Section

##### Differentiation of Human Induced Pluripotent Stem Cell (iPSC) to NPCs

All stem cell procedures were approved by Stanford's Stem Cell Research Oversight committee (SCRO: 616). Human iPSCs were generated from BJ fibroblasts using mRNA reprogramming factor sets leading to the overexpression of OCT4, SOX2, KLF4, and c‐MYC. Culture of the human iPSC line was carried out on a matrigel‐coated 6‐well plate in mTeSR. Cells were incubated at 37 °C in 5% CO_2_, and passaged every 5–7 d with Accutase (Innovative Cell Technologies, San Diego, CA).^[^ ^56^ ^]^ iPSCs from passage 51–55 were used in these studies.

Human iPSC‐derived NPCs were generated using defined conditions with minor modification to previously reported protocols.^[^ ^57^, ^58^ ^]^

##### NPC Differentiation Base Medium Formulation

Dulbecco's modified Eagle medium (DMEM)/F12 (50%), Neurobasal (50%), N2‐MAX (1%), B27 (1%) nonessential amino acids (NEAA) (1%), GlutaMAX (1%), 2‐mercaptoethanol (0.1 × 10^−3^ m), and penicillin/streptomycin (P/S, 1% v/v) were supplemented with dual SMAD inhibitors such as Dorsomorphin (1 × 10^−6^ m) and SB431542 (1 × 10^−6^ m).

##### NPC Maintenance Base Medium Formulation

DMEM/F12 (50%), Neurobasal (50%), N2‐MAX (1%), NEAA (1%), GlutaMAX (1%), 2‐mercaptoethanol (0.1 × 10^−3^ m), P/S (1% v/v) were supplemented with bFGF (20 ng mL^−1^) and EGF (20 ng mL^−1^).

##### Day (0)

Human iPSCs at ≈90% confluency were first washed with room temperature 1X Dulbecco's phosphate‐buffered saline (DPBS) without Ca_2_^+^ and Mg_2_^+^ once. The wash was aspirated and cells were primed by the treatment with NPC differentiation base medium for 7 d (4 mL per 6‐well) under standard cell culture conditions (37 °C, 5% CO_2_). Fresh medium was replenished every 24 h.

##### Day (7)

After the induction procedure, NPCs were washed with DPBS once. The cells were then detached from the plates with Accutase (1 mL per well) and incubated (37 °C). After 5 min, the side and bottom of the plate was gently rubbed to dislodge the cells from the plate surface. Then cells were collected into a 15 mL conical tube using a 10 mL serological pipette and 9 mL of DMEM/F12 containing RhoA/ROCK inhibitor, TV (2 × 10^−6^ m), was added. Cells were centrifuged at 1200 rpm for 5 min at room temperature. After centrifugation, the supernatant was aspirated and the cell pellet was resuspended in NPC maintenance medium + TV (2 × 10^−6^ m). Cells were re‐plated on 6‐well plates previously coated with Matrigel (100 000 cells cm^−2^). Then, the plate with the cells was incubated under standard cell culture conditions (37 °C, 5% CO_2_) for 24 h.

##### Single‐Cell Encapsulation of NPCs via Click Chemistry

NPCs plated on 6‐well plates were maintained with NPC maintenance media. For the in vitro experiment, NPCs at 80% confluency were treated with Ac_4_ManNAz (10 × 10^−6^ m) (Kerafast, Boston, MA) for 2 d. The cells were washed with PBS and trypsinized from the plates with Accutase. The cells were collected by centrifugation (1200 rpm for 5 min) and resuspended in the media containing different molecular weights of DBCO‐PEG (5, 10, 20, and 30 kDa; 100 000 cells mL^−1^) (BroadPharm, San Diego, CA) at a concentration of 1 µg mL^−1^ for 1 h at 37 °C. Subsequently, the cells were rinsed with PBS and resuspended in NPC maintenance media.

##### Optimization of Single‐Cell Encapsulation of NPCs

The optimal parameters including concentration of polymer and incubation time were investigated using DBCO‐PEG‐Cy5. After cell encapsulation with different parameters, the fluorescent intensity of the media containing cells was read by a multiplate reader (SpectraMax, Molecular Devices, CA) (Ex: 535 nm; Em: 585 nm). In addition, the cells treated with varying concentration of DBCO‐PEG‐Cy5 at constant incubation time of 1 h were imaged using fluorescent microscope (Keyence BZ‐X700E, Itasca, IL). Controls were 1) cells treated only with Ac_4_ManNAz (without polymer coating; 0 ng mL^−1^ of polymer) and 2) cells incubated with 1 µg mL^−1^ of DBCO‐PEG‐Cy5 without prior treatment with Ac_4_ManNAz. From the optimization study, the optimal concentration of polymer (1 µg mL^−1^) and incubation time (1 h) were utilized for further analysis.

##### Viability Assay

The viability of cells encapsulated with varying molecular weight of DBCO‐PEG was evaluated using Alamar Blue assay and Live/Dead staining. For Alamar blue assay, a 10% Alamar blue cell viability reagent was added to each sample and incubated at 37 °C for 3 h in the dark. The experimental groups were cells encapsulated with different molecular weight PEG and the controls were 1) cells without encapsulation (C), 2) cells incubated with 1 µg mL^−1^ of DBCO‐PEG (30 kDa) without prior treatment with Ac_4_ManNAz (#), 3) cells treated with Ac_4_ManNAz alone without any polymer (0 kDa), and 4) cell incubated with cell lysis buffer for 1 h (negative control, ‐). After 3 h of incubation, the absorbance of about 100 µL per sample were measured in duplicates at 570 and 600 nm using a multiplate reader (SpectraMax, Molecular Devices, CA). The percentage reduction in absorbance (percentage viability) was calculated with respect to control—cells without encapsulation as per the manufacture protocol. For Live/Dead staining, the samples were incubated with 2 µL mL^−1^ of ethidium homodimer‐1 and calcein AM for about 15 min at 37 °C in the dark. After incubation, the cells were rinsed with 1X PBS, and imaged using a fluorescent microscope (Keyence BZ‐X700).

##### Transmission Electron Microscopy

The morphology of the PEG (30 kDa) coated NPCs synthesized at optimized parameters were characterized using a TEM (JEM‐1400, Peabody, MA). Briefly, the samples were fixed with 4% paraformaldehyde in 1X PBS for 1 h at room temperature, washed thrice in 1X PBS, re‐suspended in gelatin for 5 min and cut into blocks. The blocks were post‐fixed with osmium tetroxide and uranyl acetate, serially dehydrated with ethanol, and embedded in Epon. Ultrathin sections of the samples were sliced and examined using the JOEL–JEM 1400 TEM operated at 120 kV and the images were captured using a Gatan Orius 10.7 megapixel CCD camera. The images were processed to enhance the contrast using the Adobe Photoshop.

##### AFM Force‐Distance Elasticity Measurements

Force‐distance (FD) measurements of cells attached to round glass cover slips coated with matrigel were performed in a liquid cell. Measurements were taken either using a Park NX‐10 AFM (Park Systems, Santa Clara, CA) and the temperature was maintained at 37 °C throughout the experiment. Tips with a silicon oxide spherical indenter (1 µm radius, *k* = 0.08 N m^−1^ as reported by the manufacturer, verified by a thermal tune calibration) were used on individual cells (NanoAndMore USA, Lady's Island, SC). Each cell was probed two times and a total of 20 cells was measured. Young's moduli were calculated with SPIP software (Image Metrology, Hørsholm, Denmark), which used the Hertz model for spherical indenters to fit the approach curve.

##### RNA Isolation and Quantitative Real‐Time Polymerase Chain Reaction (qRT‐PCR) Analysis

The transcription of trophic factors were measured using qRT‐PCR. Total RNA was extracted from cells using a Qiagen RNeasy Plus Micro Kit (Qiagen, Germantown, MD). After accomplishing first‐strand cDNA synthesis by iScript cDNA Synthesis Kit (Bio‐Rad, Hercules, CA), qRT‐PCR was performed with TaqMan‐polymerase and primers (Qiagen, Germantown, MD) for gene expression analysis. Pre‐developed TaqMan reagents were used for human VEGFA (Hs00900055_m1), BDNF (Hs02718934_s1), NRN1 (Hs02786624_g1), CNTF (Hs00905042_m1), ADCY8 (Hs00905042_m1), and housekeeping gene was GAPDH (Hs02786624_g1). qRT‐PCR was carried out on a QuantStudio 6 Flex Real‐Time PCR System (ThermoFisher, Waltham, MA). The Delta‐Delta CT method was utilized for relative expression levels with GAPDH as a housekeeping gene and iPSCs grown on glass as references.

##### ELISA Analysis

For VEGFB ELISA, the conditioned media was collected at 24 h after single‐cell encapsulation. Controls were cells without polymer encapsulation (C). The supernatants were collected for ELISA analysis. Samples were assayed by the VEGFB Development kit from Peprotech (Peprotech, Rocky Hill, NJ) according to the manufacturer's instructions.

##### cAMP Measurement

cAMP levels in cells were measured using the cAMP‐Glo Assay (Promega, Madison, WI). Briefly, encapsulated‐cell pellets were collected by centrifugation and treated with cAMP‐Glo lysis buffer (20 µL). The lysis solution was kept with shaking at room temperature for 15 min. After the lysis process, the cAMP detection solution was added into lysis solution (40 µL) and mixed by shaking for 1 min. The solution was further incubated at room temperature for 20 min. After the incubation, Kinase‐Glo reagent was added into the solution and incubated at room temperature for 10 min. The luminescence of the samples was measured with a plate‐reading luminometer (SpectraMax, Molecular Devices, CA).

##### Inhibition Study

After cell encapsulation with two different molecular weight polymers such as 5 and 30 kDa, the cells were rinsed and resuspended in the media containing actin polymerization inhibitor (CytoD: 2 × 10^−6^ m)^[^ ^48^, ^49^ ^]^ and activator (lysophosphatidic (LPA): 0.5 × 10^−6^ m).^[^ ^50^ ^]^ After the incubation for 24 h with different pharmacological chemicals, the supernatants from different treatment groups were collected to measure VEGFB production from the cells using ELISAs as above.

##### Statistical Analysis

All the data are presented as the mean ± standard deviation (S.D.) of four independent experiments (biological replicates). *n* values indicate the number of independent experiments conducted or the number of individual experiments. An analysis of variance (ANOVA) test was used for multicomponent comparisons (*n* > 3 independent variables) after the normal distribution was confirmed. Tukey post hoc analysis was performed to investigate the differences between variables.

## Conflict of Interest

The authors declare no conflict of interest.

## Supporting information

Supporting Information
