# Supplementary material for: Single‐Cell Encapsulation via Click‐Chemistry Alters Production of Paracrine Factors from Neural Progenitor Cells
Source: Adv Sci (Weinh). 2020 Mar 5;7(8):1902573. doi: 10.1002/advs.201902573 (PMC7175248; doi:10.1002/advs.201902573)
Supplement: Supplementary file 1 — Supporting Information [file ADVS-7-1902573-s001.pdf]

Copyright WILEY-VCH Verlag GmbH & Co. KGaA, 69469 Weinheim, Germany, 2020.

ADVANCED SCIENCE
Open Access

Supporting Information

for *Adv. Sci.,* DOI: 10.1002/advs.201902573

Single-Cell Encapsulation via Click-Chemistry Alters Production of Paracrine Factors from Neural Progenitor Cells

*Byeongtaek Oh, Vishal Swaminathan, Andrey Malkovskiy, Sruthi Santhanam, Kelly McConnell, and Paul M. George**

Supplementary Material

Title: Single-cell Encapsulation via Click-chemistry Alters Production of Paracrine Factors from Neural Progenitor Cells

Byeongtaek Oh[1], *Vishal Swaminathan*[1], *Andrey Malkovskiy*[2], *Sruthi Santhanam*[1], *Kelly McConnell*[1], *Paul M. George*[1,*]

Dr. B. Oh, V. Swaminathan, Dr. S. Santhanam, K. McConnell, Prof. P.M. George

Department of Neurology and Neurological Sciences, School of Medicine, Stanford University Stanford, CA 94305, USA

E-mail: pgeorge1@stanford.edu

Dr. A. Malkovskiv

Biomaterials and Advanced Drug Delivery Laboratory, School of Medicine, Stanford University Stanford, CA 94305, USA

*Correspondence to:

Paul M. George, MD, PhD
Assistant Professor, Department of Neurology and Neurological Sciences
300 Pasteur Dr., MC5778
Stanford Stroke Center, School of Medicine
Stanford University
Stanford, CA 94305-5778
Tel: +1 (650) 723-5072
Email: pgeorge1@stanford.edu

Key words: extracellular matrix, Single cell encapsulation, ADCY8-cAMP,

Mechanotransduction, glycoengineering

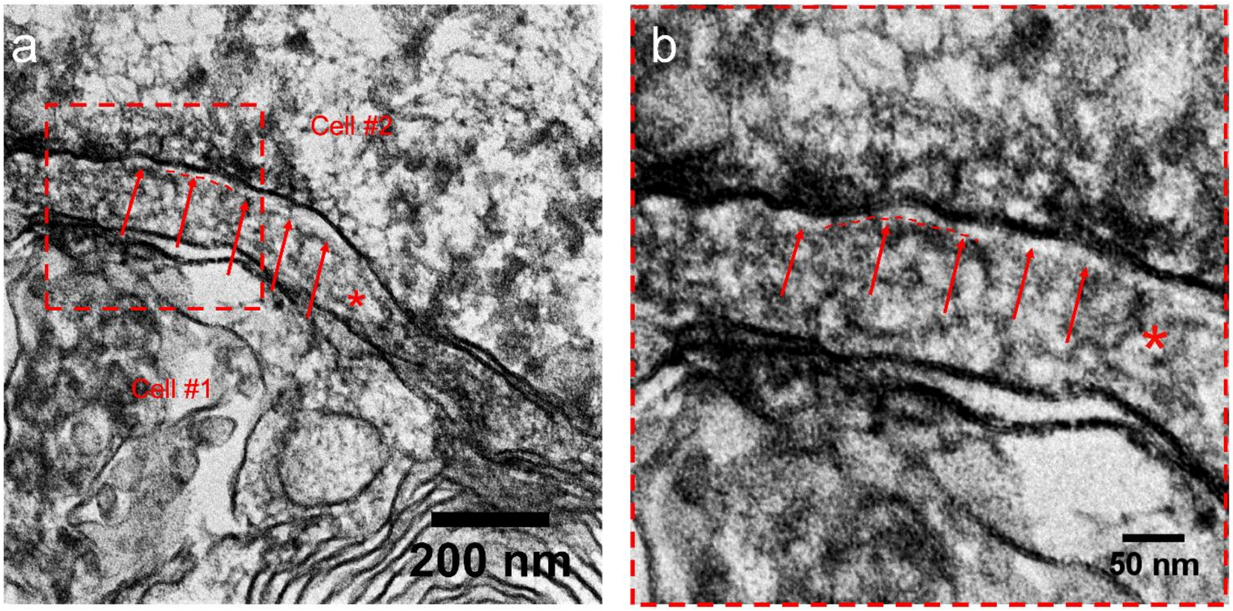

Supplementary Figure 1: TEM image of a PEG (30 kDA) coated cell. The arrows might trace a layer of polymer along the cell membrane in the intercellular space (*) between two cells. b: a higher magnification image of a section enclosed in figure a (dotted box).

To ascertain that individual cells were indeed being coated, the cells were visualized with transmission electron microscopy (TEM, JEM-1400, JEOL solutions, Peabody, MA) and fluorescent microscopy (BZ-X710, Keyence, Itasca, IL). The TEM image of a 30 kDa PEG coated NPC sample show a layer of different grayscale along the cell membrane (**Supplementary Figure 1a, b**). As the cell diameter is larger (μm range) compared to the 2-3 nm of polymer coating around the cell, the coating was not distinctly visible in the lower magnification images. Figure 1a shows a slightly lower magnification image that shows the cells partially along with a difference in grayscale that might trace a layer of polymer coating extracellular to the cell membrane. The arrows along the different grayscale suggest a layer of polymer coating around the cell. To verify a layer of polymer surrounding individual cells, high magnification images of fluorescently-tagged PEG were obtained. The images reveal a layer of red fluorescence around the NPC with the Ac_4ManNAz moiety, confirming a single-cell nano-encapsulation with the FL 545-PEG (**Figure 3b**).
